# New or repurposed: a novel classification system for the horizon scanning of innovative medicines

**DOI:** 10.1017/S0266462324004628

**Published:** 2024-12-09

**Authors:** Ross Fairbairn, Sola Akinbolade, Diarmuid Coughlan, Dapo Ogunbayo, Nick Meader, Dawn Craig

**Affiliations:** 1 National Institute for Health and Care Research (NIHR) Innovation Observatory, Population Health Sciences Institute, Faculty of Medical Sciences, Newcastle University, Newcastle, UK; 2 Population Health Sciences Institute, Faculty of Medical Sciences, Newcastle University, Newcastle, UK

**Keywords:** classification, pharmaceutical preparations, database, drug approval

## Abstract

**Objectives:**

It is vital that horizon scanning organizations can capture and disseminate intelligence on new and repurposed medicines in clinical development. To our knowledge, there are no standardized classification systems to capture this intelligence. This study aims to create a novel classification system to allow new and repurposed medicines horizon scanning intelligence to be disseminated to healthcare organizations.

**Methods:**

A multidisciplinary working group undertook literature searching and an iterative, three-stage piloting process to build consensus on a classification system. Supplementary data collection was carried out to facilitate the implementation and validation of the system on the National Institute of Health and Care Research (NIHR) Innovation Observatory (IO)‘s horizon scanning database, the Medicines Innovation Database (MInD).

**Results:**

Our piloting process highlighted important issues such as the patency and regulatory approval status of individual medicines and how combination therapies interact with these characteristics. We created a classification system with six values (New Technology, Repurposed Technology (Off-patent/Generic), Repurposed Technology (On-patent/Branded), Repurposed Technology (Never commercialised), New + Repurposed Technology (Combinations-only), Repurposed Technology (Combinations-only)) that account for these characteristics to provide novel horizon scanning insights. We validated our system through application to over 20,000 technology records on the MInD.

**Conclusions:**

Our system provides the opportunity to deliver concise yet informative intelligence to healthcare organizations and those studying the clinical development landscape of medicines. Inbuilt flexibility and the use of publicly available data sources ensure that it can be utilized by all, regardless of location or resource availability.

## Background

Horizon scanning for innovative medicines is a process for the systematic identification of medicines in clinical development to provide early awareness to policymakers and stakeholders, enabling more efficient adoption of innovations (1). The National Institute for Health and Care Research (NIHR) Innovation Observatory (IO) undertakes horizon scanning for innovative medicines to inform leading healthcare organizations in the UK, including the National Institute for Health and Care Excellence’s (NICE) health technology assessment (HTA) operations. As an unintended consequence of the COVID-19 pandemic, policymakers involved in HTA now have an interest in whether innovative medicines are new or repurposed, to make prioritization decisions and gain efficiencies in their work programmes.

It is vital that horizon scanning organizations can provide concise, yet informative intelligence to healthcare stakeholders. The volume of novel, innovative medicines being developed has increased (2), putting strain on horizon scanning resources to match the increasing activity. Furthermore, medicines repurposing, a process broadly defined as identifying new targets for medicinal products outside of the scope of the indication for which they were initially approved (3), has emerged as an area of significant interest in the clinical development landscape for researchers and policymakers. This interest is evidenced in new initiatives such as the Medicines Repurposing Programme (MRP) created by NHS England (4) and the New Therapeutic Uses program at the National Center for Advancing Translational Sciences (NCATS) in the United States (5). With these developments in mind, horizon scanners require a system to classify medicines and technologies according to whether they are new or repurposed, while accounting for important variables within those groups, including combination therapies and issues of patency.

There is a lack of existing horizon scanning methodologies to facilitate the classification of new or repurposed medicines in development. This may be owing to the field of medicines repurposing suffering from a lack of standardization in nomenclature, where numerous synonyms (e.g. repositioning, reprofiling, and redirecting) are commonly presented, and no definitive regulatory definition exists (6). As a result, we set out to create a novel classification system for the horizon scanning of innovative medicines.

Due to the relative paucity of relevant and similar systems, we focused our creative approach on the needs of the IO’s horizon scanning and utilized a multidisciplinary working group that aimed to build a system through consensus building and piloting exercises. The IO uses an internal horizon scanning database, the Medicines Innovation Database (MInD). MInD holds data on innovative new and repurposed medicines that are in active clinical development. Intelligence from MInD is provided to many health care systems and policymakers in the UK, including the National Institute for Health and Care Excellence (NICE) and the Medicines and Healthcare products Regulatory Agency (MHRA). While we utilized IO data in this work, we aimed to create a system that can provide insights for any horizon scanning conducted for innovative new and repurposed medicines, by ensuring our classification was not specific to IO needs and utilized publicly available data sources.

## Methods

In July 2021, a multidisciplinary working group was formed of four researchers (RF, SA, DC, DO) from the IO and Newcastle University. The team was comprised of members with skills and expertise from diverse backgrounds including medicines horizon scanning, pharmacy, literature searching, evidence synthesis, and HTA. The remit of the working group was to create a novel classification system for innovative new and repurposed medicines in clinical development that are identified in horizon scanning activities. This classification system was then applied to the IO’s bespoke horizon scanning database MInD, to capture and disseminate intelligence on innovative medicines in clinical development (7). Individual entries on the MInD are referred to as “technologies” (or “technology records”), a term which is used to encompass both the medicinal product(s) and the target patient group that the product(s) are being studied for in clinical trials. Technology data in MInD are taken from numerous horizon-scanning sources including trial registries and news media platforms.

### Classification development

#### Literature searching and initial classification system.

A targeted, nonsystematic search was conducted for any existing classification systems that would suit the data structure of MInD and provide the level of rigor and quality required for horizon scanners. Journal articles, grey literature, regulatory agency websites, and medical information repositories were searched to collate all relevant information, including nomenclature currently in use to refer to new medicine development and medicines repurposing. We searched for instances of classifications being applied to innovative medicine development, medicine repurposing and approval of new and repurposed medicines. This information assisted in framing our conversations and assessing existing systems and their relevance to horizon scanning. The group met weekly while searching was undertaken, to assess the literature and the data needs of the MInD, which led to a preliminary classification being proposed. Subsequent meetings were used to begin an iterative piloting process, which facilitated the creation of a novel draft classification system.

The team aimed to refine the draft classification system by consensus building and piloting. This process was formed of three rounds of piloting where the team manually applied the draft classification to existing MInD technology records and used examples to pilot the system. Using an iterative process, additional categories were added to the system based on needs identified during the pilot.

After finalizing the classification, some of the working group (RF, SA, DO) began working on the implementation of the system into MInD. Development work was conducted in conjunction with a third-party software developer. Due to the large volume of technologies stored in the MInD, we underwent a one-time, data-driven import of values for the Technology Type field. To inform the automation of the import, we created an algorithm and carried out supplementary data collection (see below).

### Supplementary data collection

The classification required three important data points to inform the algorithm: Medicines and Healthcare products Regulatory Agency (MHRA) Marketing Authorisation (MA) data from the Electronic Medicines Compendium (EMC) (8), Food and Drug Administration (FDA) Abbreviated New Drug Applications (ANDA) data from the Orange Book (9), and intervention data identifying whether the intervention(s) in the technology were being tested as a monotherapy or in combination. An existing MInD field (Intervention Type) contained data tagging each technology as Monotherapy or Combination, based on the clinical trial(s) which informed the technology. Supplementary data collection was required for the remaining two data points.

A data table of all interventions listed on EMC was exported from the EMC web page interface. We used the medicine name of interventions to match with intervention names on MInD. We considered the presence of a Summary of Product Characteristics (SmPC) on EMC as evidence of a valid MA in the UK. If a medicine name or alias matched to a MInD intervention, an existing intervention field on MInD titled Licensed in the UK was assigned the value Yes. Any unmatched MInD interventions were assigned the value No.

FDA ANDA data was extracted from an FDA data source using the Orange Book Data Files (9). We used the intervention names from the Orange Book data to match with intervention names on MInD. Per the FDA guidance in the Orange Book Preface section 1.7, we considered a medicine to be off-patent (that is to have an approved generic equivalent) when any single entry for that medicine was assigned a TE Code containing the letter A (10). If a medicine matching this criterion was matched to a MInD intervention, an existing field on MInD titled Generic/Off-patent was assigned the value Yes. Any unmatched MInD interventions were assigned the value No.

## Results

### Piloting stage one

Following initial literature searches, we started piloting technology records with a system which utilized two values: New Technology and Repurposed Technology. We defined New Technology as a technology that only contains medicine(s) that do not hold a valid Marketing Authorisation (MA) in the UK. Alternatively, a Repurposed Technology was defined as a technology that contains medicine(s) which hold at least one valid MA in the UK.

The first stage of piloting identified challenges with classifying several combination therapies, which included medicines with conflicting MA statuses. For example, Pulrodemstat (no MA in the UK) in combination with Nivolumab (MA in the UK) in patients with advanced cancers (11). Our initial system was unable to classify these types of combination treatments into one unique category. Adjustments were made to the system to incorporate new values able to adequately reflect technologies containing medicines with conflicting MA statuses in combination therapies. The two resulting additional values were “New + Repurposed Technology [Combinations-only]” and “Repurposed Technology [Combinations-only].”

### Piloting stage two

We continued the piloting process with three values in the classification. Further testing against MInD technologies, combined with our wider understanding of medicines regulation, raised the issue of patency and marketing exclusivity status. A patent is defined as a legal instrument which provides the holder of the patent the exclusive right to exclude others from making, using, selling or offering a product (12). A patent on a medicine provides the patent holder with exclusive rights to market the medicine for a limited number of years, at which point a patent will expire and others are legally able to create and market the same medicine (12). Exclusivity refers to a similar period of marketing exclusivity for a medicine, but is granted and maintained by regulatory agencies. Medicines may be granted extended periods of exclusivity based on characteristics such as Orphan Drug Designations or the medicine targeting a pediatric indication (13). The two share similarities and work in tandem to provide protection against competition for medicine for limited periods (14). As it is an area of significant complexity, we decided to treat patency and exclusivity as interchangeable, to avoid introducing unnecessary complexity into our system.

This created a distinction between technologies we classified as a Repurposed Technology. Medicine(s) in a Repurposed Technology could be on-patent/on-exclusivity (covered by patency protections or market exclusivity, often referred to as brand-name or branded) or off-patent/off-exclusivity (patency protections and market exclusivity have expired, often referred to as generic) (15). This distinction is important intelligence in a horizon-scanning context, as the patency/exclusivity status of technologies can be highly relevant to decision-making for health systems and policymakers. Generic medicines are associated with huge potential savings in health systems (15) and organizations such as the NHS England MRP have a particular interest in repurposed technologies that are off-patent (4). As a result, the team split the Repurposed Technology value into two new values, “Repurposed Technology [Off-patent/Generic]” and “Repurposed Technology [On-patent/branded].”

### Piloting stage three

The team undertook a third stage of piloting with five values of classification. At this stage, we identified a subset of technologies which could be considered repurposed but did not align with the definition of repurposing initially adopted. These technologies contained medicine(s) that have been through traditional clinical development pathways and failed in clinical trial(s). These medicines can then be acquired by another developer in a process of in-licensing (16) and begin a new clinical development program in a different indication.

This issue can be illustrated with another example of a technology based on the clinical trial NCT03263026 (17), which tested enzastaurin for diffuse large B-cell lymphoma (DLBCL) in patients with Denovo Genomic Marker 1 (DGM1). Enzastaurin was initially developed by Eli Lilly but failed to demonstrate efficacy in the phase three PRELUDE clinical trial for DLBCL in 2013. Development of enzastaurin was subsequently discontinued by Eli Lilly (18). Denovo Biopharma acquired enzastaurin in 2014 (19) and began clinical testing in new target patient groups outside of those targeted by Eli Lilly, including the subgroup of DLBCL patients targeted in NCT03263026 (17).

The working group agreed that these instances should be considered as a process of repurposing. However, the current version of the classification system would categorize this technology as a New Technology because enzastaurin did not hold a valid MA at any stage. Consequently, a sixth category, Repurposed Technology [Never Commercialised], was incorporated into the draft system.

Further piloting and *ad hoc* searching of the MInD were carried out and a consensus was reached that all important elements of repurposing and regulatory approval had been incorporated, while no further examples of technologies that did not fit the system were being found. At this stage, a final classification system (Table 1) was confirmed. Following confirmation of our final classification system, we created a new field on MInD to hold the new value named Technology Type. Our classification values were applied to this field.

**Table 1.** Technology Type values and descriptions of their application

Technology Type value	Description
New Technology	Medicinal product(s) not licensed for any indication in the UK that is a new chemical entity (NCE) or new active substance (NAS).
Repurposed Technology [Off-patent/Generic]	Medicinal product(s) are based on the same chemical structure of the therapeutically active ingredient as the original medicine which is already licensed for a different indication/target population in the UK. Off-patent refers to the medicine being generic, and any periods of patency protection have expired. Off-exclusivity refers to the medicine no longer being in any exclusive marketing period.
Repurposed Technology [On-patent/Branded]	Medicinal product(s) are based on the same chemical structure of the therapeutically active ingredient as the original medicine, which is already licensed for a different indication/target population. On-patent refers to the medicine being considered ‘branded’, as it remains under a period of patency protection.
Repurposed Technology [Never commercialised]	Medicinal product(s) that have previously been in failed clinical development where a license was not sought, for reasons including poor safety and/or efficacy profiles or a lack of commercial will to seek a license. These medicines have then been picked up again by another organization and often tested in different indications.
New + Repurposed Technology [Combinations-only]	Medicinal products being studied in combination therapies where at least one of the medicines is not licensed for any indication in the UK and at least one of the medicines is based on the same chemical structure of the therapeutically active ingredient as in the original medicine, which is already licensed for a different indication/target population in the UK.
Repurposed Technology [Combinations-only]	Medicinal products are being studied in combination therapies where all medicines are based on the same chemical structure of the therapeutically active ingredient as in the original medicine which is already licensed for a different indication/target population in the UK.

### Application of the system to the MInD

**Figure 1.**
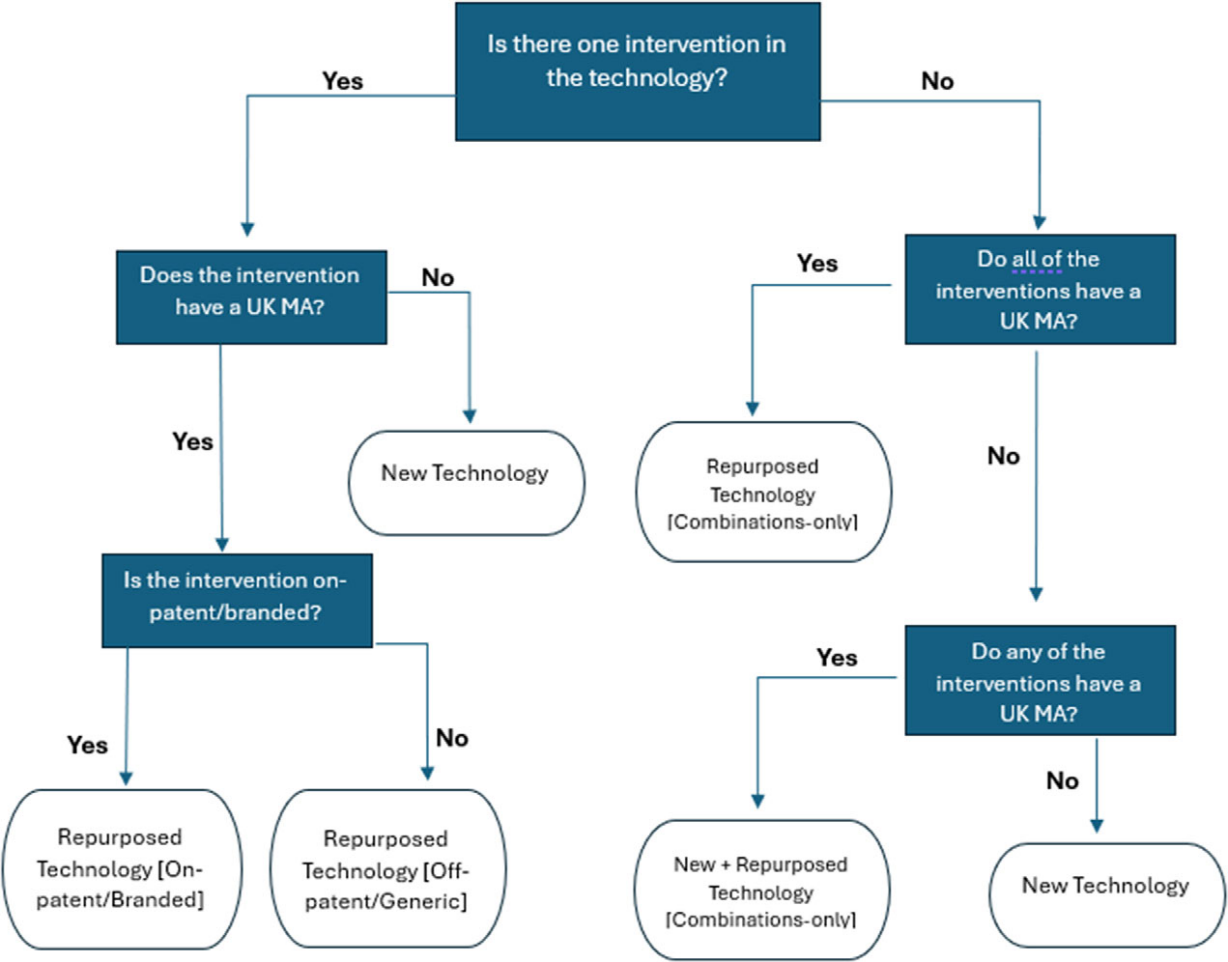
A decision chart for the import of values in Technology Type field on MInD. UK = United Kingdom, MA = Marketing Authorisation.

An import algorithm was created that utilized these three data points and an iterative, decision-based approach to set values during a one-time import, facilitated by MInD software developers. Any subsequently created technology records after the import were to be manually classified by MInD users. A diagrammatical representation of the import algorithm is presented in Figure 1. Note that the Repurposed Technology [Never Commercialised] value is not included here, as it is only set manually by MInD users.

As a further validation of the system, in January 2022, 20,403 technologies included in MInD were assigned to one of these six categories (see Table 2).

**Table 2.** Technology Type values for all MInD technologies (n = 20,403) following an initial data import

Technology Type value	Technologies assigned to (%)
New Technology	10,396 (51.0%)
Repurposed Technology [Off-patent/Off-exclusivity]	1,628 (8.0%)
Repurposed Technology [On-patent/Branded]	3,192 (15.6%)
Repurposed Technology [Never commercialised]	2 (0.01%)
New + Repurposed Technology [Combinations-only]	3,034 (14.9%)
Repurposed Technology [Combinations-only]	2,151 (10.5%)

10,396 (fifty-one percent) of technologies were categorized as a New Technology, while 6,973 (thirty-four percent) were assigned to one of four categories of repurposed technology. 3,034 (fifteen percent) were assigned to the New + Repurposed Technology [Combinations-only] category.

## Discussion

In this study we have created, to our knowledge, the first system that classifies innovative new and repurposed medicines for comprehensive medicines horizon scanning activities. We have built and validated our classification system on a living horizon scanning database with over 20,000 technologies. The system offers those studying the clinical development landscape of medicines the opportunity to collect succinct, yet informative intelligence for individual technologies on important issues such as medicine repurposing, regulatory approvals, patency, and combination therapies.

The values presented here make the important distinction between patented and generic medicines within the repurposing umbrella. This distinction is important as these two groups of repurposed medicines are likely to be subject to differing repurposing opportunities and will follow unique regulatory paths; therefore, it is important that healthcare organizations are aware of this (20). Additionally, the classification accounts for the complexity of combination therapy developments, while still retaining some separation between new and repurposed medicines within combinations. This allows horizon scanners to report insights which account for potential combinations of medicines, which are otherwise difficult to categorize.

Our iterative piloting system using MInD technologies meant that our classification was created based on real-life examples from a leading medicines horizon scanning database. The large volume of technologies in the database ensures that it is unlikely we have missed any distinct classifications.

The introduction of this novel classification system has improved the IO’s capability to disseminate high-quality horizon scanning intelligence to its stakeholders and is now in routine use on the MInD. The Technology Type field is used to inform NICE topic selection and HTA operations through intelligence outputs created for medicines by the IO. Additionally, the field now forms a vital element of horizon scanning intelligence provided to the MRP at NHS England, offering insight in to off-patent technologies which are of particular interest to the program. Finally, the field contributes to intelligence provided on interactive dashboards hosted by the IO for public use at https://www.io.nihr.ac.uk/latest-dashboards.

One of the main challenges we faced during this project was poor availability of external data that is needed to inform the classification. We use UK MA data for medicines, but this is paired with United States FDA Orange Book data intervention patency statuses, due to a lack of publicly available data sets for UK medicine patents. While we believe that patency and exclusivity in medicines often align well from the US to the UK, there may be issues when a medicine’s patency status differs between the two countries, which could lead to misclassification of a technology. Additionally, while EMC data is a strong proxy for UK MAs, EMC only list SmPCs for medicines which have been launched on the market. This means that any approved medicines not yet launched on the market would not be captured, again leading to potential misclassification.

We believe that our classification is valid for use outside of the UK, as horizon scanners will be able to substitute regulatory approval and patency data values into the system that are more relevant to their geographies. Many international regulators including the European Medicines Agency in the European Union (21), the FDA in the United States (22), Health Canada (23), and the Therapeutic Goods Administration in Australia (24) provide routine data on medicines approvals which may be used. However, patency data in different geographies can be difficult to source, hence the use of United States data in a UK context here. Improved transparency and data-sharing policies from international regulators would enable horizon scanning systems to capture and disseminate more effective intelligence for medicines.

Our approach of a one-time classification of values based on static data points can lead to limitations when working with the data in the future. As we have no provision for updating the field, classifications can only be considered accurate at the point of initial import, or technology record creation for any subsequently created technologies. Some data driving the classification are subject to change over time, for example, a medicine being granted an MA for the first time or losing patency/exclusivity. However, the lack of real-time data updates means that the Technology Type value would not change simultaneously, potentially leading to difficulty interpreting the field for older technologies. Additionally, limitations may be introduced in our system as we were not able to work with stakeholders during the creation process. Stakeholders would be able to provide unique insights on data needs that could improve the classification system and that may be overlooked by those creating it. This should be implemented for future work in this area.

Work is currently ongoing at the IO to incorporate the classification algorithm in to MInD directly, meaning that technologies will be automatically classified at the point of record creation, thereby reducing the manual resources required for upkeep. Future work in the area should focus on novel sources for supplementary data collection. Researchers should look to advanced computing and data science methodologies to build improved automation and data collection methods to provide rigorous and insightful horizon scanning intelligence to healthcare systems.
